# Liquid–solid composites with confined interface behaviors

**DOI:** 10.1093/nsr/nwae423

**Published:** 2024-11-23

**Authors:** Shijie Yu, Yina Jiang, Lejian Yu, Huimeng Wang, Liting Pan, Jian Zhang, Yunmao Zhang, Xu Hou

**Affiliations:** State Key Laboratory of Physical Chemistry of Solid Surfaces, College of Chemistry and Chemical Engineering, Xiamen University, Xiamen 361005, China; State Key Laboratory of Physical Chemistry of Solid Surfaces, College of Chemistry and Chemical Engineering, Xiamen University, Xiamen 361005, China; State Key Laboratory of Physical Chemistry of Solid Surfaces, College of Chemistry and Chemical Engineering, Xiamen University, Xiamen 361005, China; State Key Laboratory of Physical Chemistry of Solid Surfaces, College of Chemistry and Chemical Engineering, Xiamen University, Xiamen 361005, China; Research Institute for Biomimetics and Soft Matter, Fujian Provincial Key Laboratory for Soft Functional Materials Research, College of Physical Science and Technology, Xiamen University, Xiamen 361005, China; State Key Laboratory of Physical Chemistry of Solid Surfaces, College of Chemistry and Chemical Engineering, Xiamen University, Xiamen 361005, China; Research Institute for Biomimetics and Soft Matter, Fujian Provincial Key Laboratory for Soft Functional Materials Research, College of Physical Science and Technology, Xiamen University, Xiamen 361005, China; State Key Laboratory of Physical Chemistry of Solid Surfaces, College of Chemistry and Chemical Engineering, Xiamen University, Xiamen 361005, China; Institute of Artificial Intelligence, Xiamen University, Xiamen 361005, China; Research Institute for Biomimetics and Soft Matter, Fujian Provincial Key Laboratory for Soft Functional Materials Research, College of Physical Science and Technology, Xiamen University, Xiamen 361005, China; Innovation Laboratory for Sciences and Technologies of Energy Materials of Fujian Province (IKKEM), Xiamen 361102, China

**Keywords:** liquid–solid composites, liquid-based confined interface materials (LCIMs), multiphase interfacial interactions, liquid motions, liquid gating technology

## Abstract

In the evolving landscape of materials science, the journey from traditional composite materials to liquid–solid composites has marked a significant shift. Composite materials, typically solid state, have long been the cornerstone of many applications due to their structural stability and mechanical properties. However, the emergence of liquid–solid composites has introduced a new paradigm, leveraging the dynamic composite interfaces and fluidic nature of liquids. Recent years have witnessed the rapid development of liquid–solid composites, distinguishing themselves by their defect-free, molecularly smooth surfaces and adaptive features. In this review, we introduce liquid-based confined interface materials, which represent a cutting-edge advancement, integrating confined liquids within solid frameworks at mesoscopic scales. Characterized by their confined competitive multiphase interfacial interactions, these materials offer practical functionalities like anti-fouling, multiphase flow control and drag reduction. We summarize the development of the materials, and showcase important applications based on the controllable motions of confined liquids and solid frameworks. We also discuss their design and preparation and address future challenges and outlooks, such as artificial intelligence, in advancing functionalities.

## Teaser

The review provides new insights into liquid–solid composites, focusing on their confined interface behaviors, including liquid motion and competitive multiphase interfacial interactions, and offering a pathway for future innovations and performance enhancements in liquid-based confined interface materials.

## INTRODUCTION

**Figure 1.**
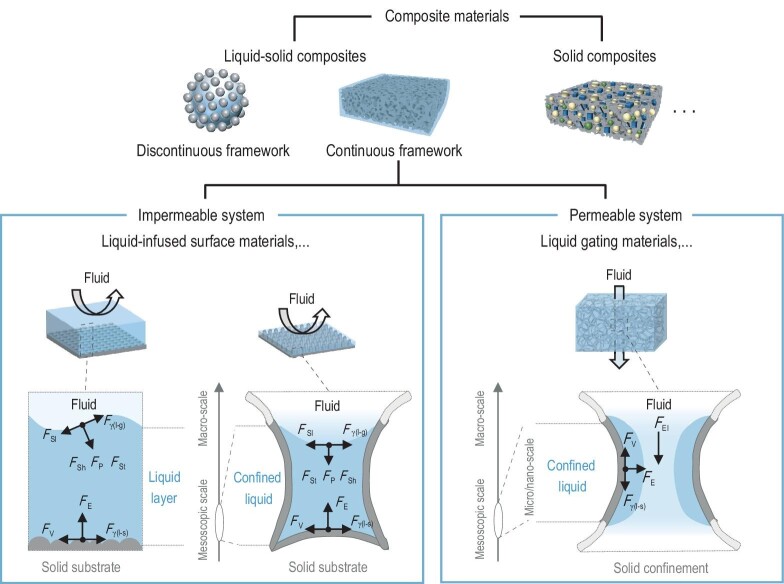
Schematic illustrations of the classification of composite materials. Composite materials can be divided into liquid–solid composites and solid composites according to their phase states. Liquid–solid composites can be categorized, based on the continuity of their solid frameworks, into those with discontinuous frameworks and those with continuous frameworks. Liquid–solid composites with discontinuous frameworks include liquid marbles and Pickering emulsions. Liquid–solid composites with continuous frameworks contain impermeable systems and permeable systems. For instance, liquid-infused surface materials represent a typical impermeable system. The external impact forces induced by fluids, such as slide force (*F*_Sl_), shear force (*F*_Sh_), pound force (*F*_P_) and strike force (*F*_St_), act on the surface of liquid layers. These forces usually significantly exceed the interaction effects felt at liquid–solid interfaces. The interactions at liquid–solid interfaces include interfacial tension (*F*_γ_), capillary force (*F*_C_), electrostatic force (*F*_E_), van der Waals force (*F*_vdW_) and viscous resistance (*F*_V_). In permeable systems, the class of liquid gating materials is a representative example, in which the fluids can penetrate through the interior of the liquids, resulting in the external impact forces (*F*_EI_) caused by fluids and interactions at liquid–solid interfaces mainly occurring at the same scale.

It is commonly thought that composite material systems usually consist of solid-state composites, and fluids have long been kept out of the selection spectrum because of their complex dynamics, fluidity, difficulties in processing, etc. [1] Nowadays, as a result of the increasing need for new dynamic and intelligent concept materials, and the advances made in the sophisticated control of fluids, dynamic material systems composed of multiphases have attracted more and more attention. Though we use gases as raw materials in industrial applications [2,3], with the presence of solids and liquids, to some extent gases do not have a composite meaning. To differentiate it from solid composites, the composite of multiphases is based on a combination of liquid and solid, and it is thus referred to as a ‘liquid–solid composite’ [4–6] (Fig. 1, top). These composites are hoped to fuse the characteristics of both solids and liquids to achieve unique advantages such as being defect-free and molecularly smooth, and having a fast dynamic response, soft interface and structural flexibility [7–9].

With the development of liquid–solid composites, a series of definitions and names for such materials have emerged, such as liquid-based porous membranes/materials [8], liquid-infused surfaces/liquid-infused porous surfaces [10–12] and liquid-based materials [6,13], based on their functional structures, usage scenarios, types of applications, etc.—these all fall under similar materials systems. The variations in solid framework structures and compositions will induce different material behaviors in the liquid, so in order to clearly discuss such materials, here we classify the liquid–solid composites based on the continuity of their solid framework structures.

For the systems with discontinuous solid frameworks, the liquid will not be confined (Fig. 1, upper left). These systems commonly encapsulate particles within liquids or disperse them into liquids through surface tensions, van der Waals force, electrostatic interaction or capillary forces, leading to the formation of liquid marbles [14–16], Pickering emulsions [17–21], etc. In such composites, the solid and liquid components typically move in unison, presenting high fluidity and malleability, and have been applied in all-liquid 3D printing [17,18,20], soft robotics [22–24], etc.

To construct liquid–solid composites with continuous solid frameworks, fixed structures of networks, pores or channels are employed as solid frameworks to confine the liquids within them (Fig. 1, bottom). When integrating with complex micro/nano-scale networks and porous structural solid materials, the liquid's properties of shape conformity led it to divide into tiny structures depending on the geometry and spacing of the confinement. These resultant confined interfaces of the liquid–solid composites, mostly within the micro-scale, are extremely sensitive to capillary force, electrostatic interaction, van der Waals force and osmotic force, and can be thought of as analogies to microfluidic systems, where the movements of liquids are confined by solid microchannels. Similarly, precise control of liquid motion can be achieved through the regulation of confined interface behaviors [25–27]. However, microfluidics is not considered suitable for utilizing liquids as long-term surface materials [28,29]. This issue can be significantly alleviated by shortening the aspect ratio of microchannels to the pore structures [30] or directly incorporating porous systems [31,32], due to the considerable increase in the proportion of liquid in these liquid–solid composites. This design offers a multitude of benefits beyond the capability to precisely regulate the fluid motion and also to amplify the influence of liquid interfaces. The continuous molecular mobility of a liquid in the confined interfaces means it is free to organize the interfaces into a molecularly smooth, homogeneous, energy-minimized state in an active process. And sufficient liquid components enable the interfaces to continuously form and regenerate [1], meaning they are more able to leverage the fluidic advantages of the materials by naturally restoring themselves through molecular turnover and local flow.

Relying on the permeability of the framework structures, the microstructures of solid frameworks may be sealed off in certain directions, limiting the connectedness between microstructures and the overall permeability of liquids in these directions. For these impermeable systems, liquid-infused surface materials are one of the typical examples (Fig. 1, lower left), leveraging the dynamics of liquids, integrated with micro/nano-structured solid substrates, and forming a continuous liquid layer along the surface of the solid substrate without permeating through the solid substrate regardless of the thickness of the liquid layers. This design of materials stabilizes the liquid to maintain an external liquid film, enhancing the durability of the systems, and can create long-term surfaces. Under the application scenario of multiphase transport control, the impermeability of the solid substrate allows the transport fluids to flow laterally along the liquid surface rather than permeate through the interior of the system, despite the external impact forces caused by the transport fluids to the liquid like shearing, pounding, striking and sliding, which usually significantly exceed the interaction effects felt at liquid–solid interfaces. The system exhibits superior properties including self-healing, anti-ice, anti-fouling and adaptability [10,33–36]. These materials have been discussed in detail, e.g. by Aizenberg J. *et al.* [1,36], Neto C. *et al.* [11], Jiang L. *et al.* [35], Howell C. *et al.* [37], Taeyoon L. *et al.* [38], Liu M. *et al.* [39] and Hou X. *et al*. [40] In addition, it is worth mentioning that there is a kind of solid composite that is coated with liquid-like flexible molecules to exhibit certain characteristics of liquid-infused surface materials [41,42].

When the solid frameworks are permeable, liquids can permeate in all directions through continuous molecular migration (Fig. 1, lower right). When these permeable systems are utilized for multiphase fluid transport, the interactions between liquids and transport fluids are also affected, while in these cases, the fluids can penetrate through the liquid surfaces or enter these confined interfaces. In contrast to the impermeable systems, here the external impact forces introduced by fluids are usually acting at the same scale as the liquid–solid interactions within. Under this scenario, if we can utilize the innate properties of liquids such as mobility to synergize the back-and-forth on the interactions between the confined liquid interface with the transport fluids and that between the liquids and their underlying porous solid interface, along with the regulations of liquids’ movements, a class of liquid–solid composites can be proposed that focus on confined interface behaviors. In fact, recent years have witnessed some advances in this direction, and the unique advantages of these materials have been explored in the areas of multiphase fluid transport control technology, offering breakthrough applications in various domains including petrochemicals [43–45], air purification [46], sewage treatment [44,47–51], pharmaceuticals [30,52], substance testing [53–55], food [56,57], cosmetics [56] and smart agriculture [58]. The emerging research on the liquid gating materials system is a representative example, and exhibits its capabilities, such as adaptivity, lack of defects, anti-fouling, self-healing, drag-reduction, optical transparency, robustness, durability, energy efficiency and biocompatibility. These material properties and performance advantages are promising, but the challenges are still significant and multifaceted, ranging from science to technological implementation. This perspective is timely because it highlights that the full potential of these materials in the field of composite materials science—particularly in the systematic design and development of such liquid–solid composites—remains largely unexplored.

**Figure 2.**
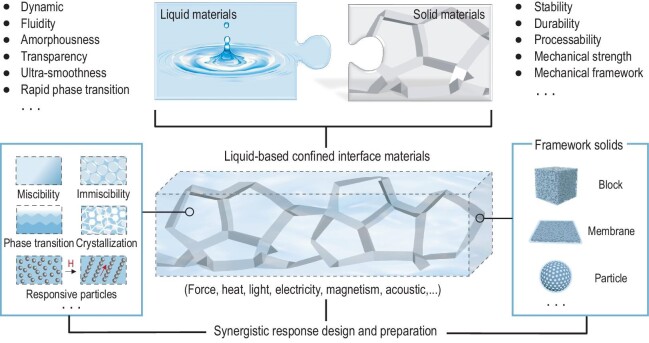
Outline of liquid-based confined interface materials and their design and preparation. Liquid materials exhibit properties, such as dynamic, fluidity, amorphousness, transparency, ultra-smoothness and rapid phase transition. Solid materials provide properties including stability, durability, processability, mechanical strength and a mechanical framework. Liquid-based confined interface materials (LCIMs) are formed by confining liquids within framework solids. Confined liquids, can be a mixture of several miscible or immiscible liquids, and they may undergo phase transition and crystallization phenomena, and contain responsive particles, etc. Framework solids can be block materials, membrane materials and particle materials, etc. Confined liquid and framework solids can be designed and prepared through synergistic responses, including force, heat, light, electricity, magnetism and acoustics.

Here we introduce the development of this class of materials, their application prospects, outlook and challenges. To start, we name these materials ‘liquid-based confined interface materials’ (LCIMs) (Fig. 2). One of the complexities with LCIMs is that, with all players within the same length scale, multiple rivalries exist to determine the materials’ functions and properties, and trivial details regarding the multiphase fluid motion behaviors at the confined interface can have niche roles in affecting the whole system. These characteristics enable LCIMs to realize new properties and functions upon interacting with multiphase fluids. Next, we will start with the design and preparation of LCIMs, highlighting the collaborative integration of the confined liquids with solid frameworks.

## Design and preparation of LCIMs

The key to developing LCIMs is the collaborative and complementary design of liquid materials and solid materials (Fig. 2, top). In general, liquid materials are characterized by dynamism, fluidity, amorphousness, transparency and ultra-smoothness, as well as rapid-phase transition, while they are not conducive to being shaped into fixed structures [7,59]. Conversely, solid materials exhibit stability, durability, processability, mechanical strength and mechanical framework properties, yet for them to achieve rapid dynamic response and defect-free interfaces remains challenging [13]. When the two are stably combined as composite materials (Fig. 2, bottom), they are characterized by the solid materials serving as frameworks, networks, pores and channels that confine the liquid materials within them. The thermodynamic stability is critical for creating LCIMs and is affected by factors such as the solubility of confined liquids and transport fluids, the interface energy and wettability between liquids and solids, and the roughness of the solid surfaces. For instance, when the transport fluid and the confined liquid are immiscible, and the interface energy between the confined liquid and the solid is lower than that between the transport fluid and the solid, it effectively ensures the stability of the materials, particularly in liquid-infused surface material systems [10,60–63] and liquid gating material systems [8,64,65]. While bridging the realms of liquid materials and solid materials, LCIMs have also expanded the material scientific problem of a single solid/liquid interface, the liquid surface, or the solid surface in the mesoscopic confinement, to a broader perspectives on the interaction and competition between multiple interfaces of solid/liquid, liquid/liquid and liquid/gas. Meanwhile, a confined liquid, with a certain defect-free material structure, can construct a soft interface to regulate the behavior of its confined superficial fluids.

**Figure 3.**
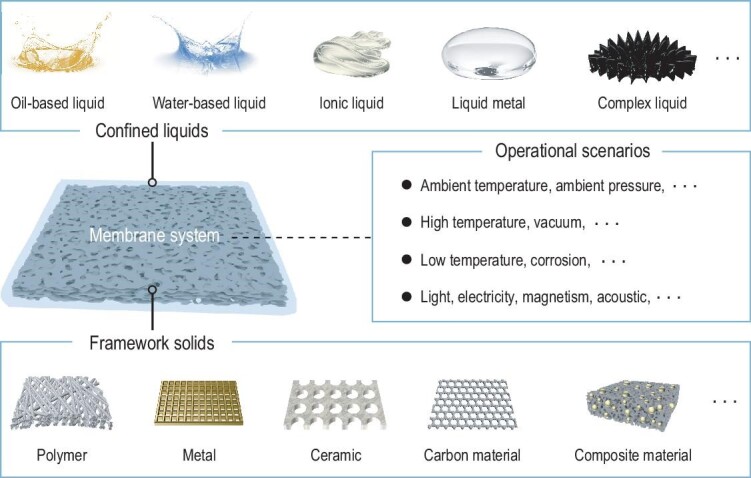
The membrane system of LCIMs. A membrane system is composed of confined liquids and framework solids. Confined liquids include oil-based liquid, water-based liquid, liquid metal, ionic liquid, complex liquid, etc. Framework solids include polymer, metal, ceramic, carbon material and composite material. According to the operational scenarios, the appropriate liquids and solids can be selected to design and prepare LCIMs.

Specifically, LCIMs can be block materials [66–70], membrane materials [8,65,71,72], particle materials [73,74], etc. Currently, the most representative system of LCIMs can be found in the area of membrane science. Among these materials, various confined liquids and solid frameworks can be selected according to the operating scenarios (Fig. 3). For instance, at ambient temperature and ambient pressure, oil-based liquid and water-based liquid, polymer, and composite materials are appropriate. However, in more extreme situations, such as high temperatures, vacuum conditions, lower temperatures or corrosive conditions, the system's interfacial durability over the long term is crucial. For these scenarios, components known for their high stability, such as metal, silicone oil and ionic liquid, are preferable to ensure enhanced system stability. Additionally, in scenarios with light, electricity, magnetism or acoustic conditions, some complex liquids contain stimuli-responsive substances, or stimuli-responsive solids are suitable, which effectively regulate the interactions between the solid and liquid phases at the confined interface, enabling the system to respond quickly and adapt to the external environment. The design and preparation of LCIMs in membrane systems have been discussed in detail [7–9,64,65] and will not be the subject of discussion in this review. But there are some latest development ideas and trends that we would like to discuss here.

For the confined liquids, controlling their movement in LCIMs is the key to realizing systematic functions. Therefore, the design of the physical and chemical properties of the liquids should no longer only take a single composition into consideration [75,76]. Confined liquids can be a mixture of several mutually soluble liquids or an emulsion of immiscible liquids. In spite of the complex properties of such liquids, it also provides more designability for the soft interface constructed by the confined liquids. On the other hand, the confined liquid phase transition behaviors in a controllable fashion can realize the regulation of softness and hardness of the confined interface. This will make some headway towards the application of multiphase fluid flow control that can withstand harsh conditions [25,46,77–80]. Even crystallization processes can be incorporated into the confined liquids, bringing more controllable approaches and realizing the regulation of confined liquid behaviors [81]. In addition, further consideration can be given to not simply utilizing the transparency of liquids. In the future, some colloidal particles can be further combined to achieve a directional arrangement of particles in the confined liquids, creating different structural colors [82], and further developing the optical application of LCIMs.

For the framework solids in LCIMs, strategies to design responsive porous solids are currently a focal research point. Equipping them with multiple external field responsiveness, including force, heat, light, electricity, magnetism and acoustics, will provide a dynamic and controllable active space for the confined liquids to further regulate the liquid motion behaviors, facilitating LCIMs with more intelligent functions. For example, using memory porous solids to provide symmetrical confined spaces has been envisioned; the porous structure undergoes memory asymmetric deformation under the action of an external field, which will lead to changes in the symmetry of the porous structure, thereby affecting the motion behavior of the confined liquid in real-time. Meanwhile, the framework solid is no longer limited to a single response system, and it can obtain a collaborative response of multiple external fields. It is worth mentioning that the responsive design is not only limited to the framework solids but can also expand to the responsiveness of the confined liquid. The responsive confined liquids, combined with unique rheological behaviors, such as the Marangoni effect, Shear thinning effect or Shear thickening effect, will bring new ideas to the functional development of LCIMs. In addition, the synergistic response design and preparation of the framework solids and the confined liquids will be an important research direction for the future development of LCIMs.

It is believed that a promising breakthrough direction for LCIMs lies in the comprehensive understanding of the dynamic liquid motion behaviors within the confined interface. For the purpose of explicitness, we would explore the motion of liquid components in LCIMs, and therefore we organize the following section according to the confined liquid motions in LCIMs, to help the reader understand.

## Liquid motion of LCIMs

**Figure 4.**
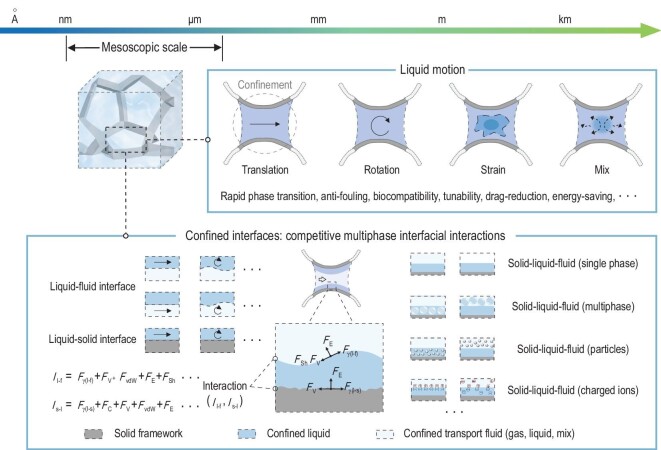
Confined liquid motion and competitive multiphase interfacial interactions of LCIMs at the mesoscopic scale. The liquid motion of LCIMs contains translation, rotation, strain, mix, etc. Liquid motion leads to competitive multiphase interfacial interactions between the liquid–solid interface and the liquid–fluid interface. There can be different kinds of composition at the solid–liquid–fluid interface, including single phase, multiphase, particles and charged ions. At the liquid–fluid interface, various forces work together to influence the interfacial interactions (*I*_l-f_), including liquid–fluid interfacial tension (*F*_γ(l-f)_), *F*_V_, *F*_vdW_, *F*_E_ and *F*_Sh_. At the liquid–solid interface, interfacial interactions (*I*_s-l_) include solid–liquid interfacial tension (*F*_γ(s-l)_), *F*_C_, *F*_V_, *F*_vdW_ and *F*_E_.

LCIMs are primarily constructed by combining multi-scale solid framework materials with liquids. The size of the solid framework dictates the dimension of the confined interface. Since the core scientific issue of LCIMs concerns different confined interfaces, in order to ensure the stable existence of liquids, the boundary size of the solid confinement space should normally be at the micrometer scale or below. On the other hand, if the solid confinement space is too small, the proportion of composite liquids will be greatly reduced, undermining the performance of LCIMs. Thus, the optimal confinement boundary condition for LCIMs is at the mesoscopic scale (Fig. 4).

Liquid motion in LCIMs refers to the mobility of the liquid component within the confined interface, but more fundamentally, what the motion actually does is allow the system to take various external driving forces to the interior of the confined interface through the media, and translate them into interaction forces, e.g. interfacial tension, capillary, shear, electrostatic and other forces that arise between multiphase transport fluid interfaces. Despite these interaction forces all acting on the same scale, with the trade-offs and reinforcements among the forces, their synergistic effects at the confined interface are complex. Due to the short distance of multiphase fluid interfaces, the minimal thickness of the liquid film brings an especially sensitive dynamic coupling between the liquid interior, the liquid–fluid interface and the liquid–solid interface. For instance, a fluid flowing through the interior of the confined liquid induces the coupled motion of the liquid–fluid interface, leading to the localized thinning of the liquid and enhancing its interplay with the solid, which changes local mobility of the liquid and further modifies its motion. Therefore, the most important features of LCIMs are that their functions are usually achieved based on the motion of confined liquids in an active process. Here we will discuss LCIMs through four representative modes of the confined liquid motion: translation, rotation, strain and mix.

For a liquid’s translational motion, where the liquid travels in a single direction along a solid or fluid surface, the fluidity of the liquid enables it to move freely over solid surfaces, resulting in a molecularly smooth liquid interface on rough solid surfaces [83]. As the translational motions continue, a liquid simultaneously moves contaminants if contained along the liquid–fluid interfaces. This effectively reduces direct contact and adhesion of pollutants to the inner surface of a solid, thereby avoiding contamination and fouling. Even highly adhering animal blood finds it extremely difficult to stick or coagulate along the internal interfaces under this constant translational motion. Studies show that the design based on LCIMs can reduce the formation of blood clots, offering anticoagulant benefits while exhibiting excellent biocompatibility [52]. Additionally, LCIMs provide efficient approaches to improving the tunability of multiphase fluid transportation. For instance, when a multiphase transport fluid contains several immiscible liquids, the competition mechanisms of interfacial tension within the multiphase fluids allow the phase with lower surface tension to slide over the liquid interface first, facilitated by its weaker intermolecular forces that enable easier outward movement. Conversely, fluids with higher surface tension do not penetrate, as their stronger intermolecular forces resist outward diffusion [83]. Moreover, drag-reduction is also built into this motion. The wettability of the liquid makes it spread into a liquid layer on the solid surface but also stabilizes the liquid layer under capillary effects, effectively avoiding direct contact between the transferring fluid and the solid. Compared to solid–fluid interfaces, the translational motion of the liquid layer facilitates the mobility of liquid–fluid interfaces, reducing the viscous resistance in fluid transmission at the interfaces. Consequently, this molecularly smooth liquid–liquid interface can notably decrease the viscous drag force, facilitating energy-saving effects. For example, the LCIM is developed for emulsification, exhibiting ultrahigh efficiency, which can achieve energy savings up to more than four orders of magnitude greater than commercial materials [56].

In addition, when further utilizing the physicochemical properties of the substances contained inside the confined liquids or the transport fluids, manifold functional LCIMs can be developed. For example, when the confined liquid contains colloidal particles with a magnetic field response, the non-contact magnetic control of colloidal aggregation behavior in the confined liquid is achieved by tuning the entropy of the colloidal suspension. The strength and direction of the magnetic field can affect the physical properties of the confined liquid, leading to an increase in viscosity and thereby enhancing the interaction between solids and liquids. The parameters, like colloidal concentration, confining ratio and external field strengths and directions, can be used to manipulate the aggregation configuration characterized by the colloidal entropy [84]. This approach can be used to tune microscale fluid transportation, and also gives us a new characterization method to study the mechanical properties of the confined colloidal suspension in real time. This strategy opens new avenues in the areas of drug delivery, microfluidic logic, dynamic fluid control, chemical reactions and more.

Likewise, when the confined liquid contains charged ions, the interfacial wettability of the solid frameworks can be controllably and reversibly changed [46]. For a transport fluid containing charged ions, the motion of the fluid induces liquid slip at the liquid–fluid interface and facilitates charge coupling, leading to an electrokinetic effect, which can be utilized to achieve a discontinuous streaming potential electrokinetic energy conversion fluid system [85]. This design creates a platform for applications in the areas of autonomous health monitoring devices, seismic sea-wave warning systems and more [85]. On the other hand, if the transport fluids are gases containing particles, the size effect would prevent larger particles in the gas from directly entering the confined space of the LCIM. After the gas comes into contact with the confined liquid, the interactions between the confined liquid interface and the transport gas, and between the confined liquid and its underlying porous solid interface, can be controlled to convert the gas into microbubbles of a controllable size [46]. The smaller the bubbles, the greater the chance for the particles inside to come into contact with the liquid to get a much higher particle absorption efficiency, due to the more efficient gas–liquid interfacial mass transfer behavior. By utilizing this property, the efficient purification of particles in the air by LCIM can be achieved. It is possible to further utilize the dynamic updates of the liquid flow in LCIMs to achieve reusability, and it is expected that a new type of consumable-free air purification system will be developed.

Beyond translational motions, the confined liquids may conduct rotational motions, where they can flow and rotate in a 3D direction along the solid or fluid surfaces. This can endow the LCIMs with more complex fluid transport control flexibility. However, achieving the confined liquids’ rotational motions in LCIMs remains challenging. In macroscopic scales, external forces such as mechanical stirring can directly cause the rotational flows of liquids, and even form vortices. These methods are not applicable for achieving controllable rotation of the mesoscopic-scale confined liquids. The essence of rotation is caused by shear stress generated by the interaction forces between molecules inside the liquids. So, it is possible to introduce certain substances into the confined liquid and achieve rotational motion through specific external non-contact effects. These substances drive the internal molecules of the confined liquid to achieve rotational motions. For example, as mentioned earlier, by incorporating magnetic colloidal particles in the confined liquid, the LCIM can conduct precise 3D microscale fluid transport behaviors through magnetic fields, enabling the confined liquid to rotate, which is caused by the rotation of contained colloidal particles [84]. It is expected that this 3D control method could combine with complex porous systems [30] in the future to achieve 3D transport control of fluids inside the LCIM.

The liquid motion of strain can lead to the deformation of a confined liquid under applied forces, which mainly come in the form of linear strain and shear strain. Linear strain refers to the elongation or compression of the liquid along a specific direction, while shear strain illustrates how parallel layers of the liquid slide past each other due to tangential forces. Similar to the translational motion, in most cases, it also relies on the substances that drive the internal molecules of the confined liquid to achieve strain motion. For instance, by utilizing the shear thickening behavior of non-Newtonian fluids, the strain motion of the confined liquid can be achieved through ultrasonic control, further enabling self-protective gas transport control of the LCIM [86]. Since there is shear thickening, shear thinning or other rheological behaviors will bring more possibilities for the LCIM's application.

At present, exploration of the mixed motion of liquids within confined interfaces is still in its early stages, and there are still many unclear scientific issues and technical challenges that remain ahead. On the one hand, the precise control of rheological behaviors of multiphase fluids is relatively difficult to achieve, especially in observing and regulating the behaviors of confined fluids at the mesoscopic scale. On the other hand, if there are issues related to the homogeneity of confined liquids or the fabrication of uniform pore sizes in porous structural solids, the factors concerning the motion of the confined liquid of the LCIMs would become even more complicated. Though challenges remain, inspiration can be drawn from the studies and models of the mixed motion behaviors of bulk liquids, to derive the effects of liquid motion within confined interfaces to some degree. For instance, in the bulk phase, diffusion [87,88] and convection [89,90] can alter the transportation of ions or particles, further affecting the charge distribution and electrostatic forces on confined interfaces. Bulk liquid's phase transition behaviors will also alter the mobility of liquid molecules [91], playing an influential role in mass transfers between phases and reaction processes. If it is introduced to the confined interfaces, the wettability and capillary forces between multiphase fluids will be altered, affecting the mass transfers and reaction processes. More importantly, sophisticated regulations on the mixed motions of liquids will realize the construction of symmetric and asymmetric multiphase fluid confined interfaces, bringing more functionalities and applications to the LCIMs.

This section discusses the importance of the mesoscopic scale for optimal confinement and explores different modes of liquid motion within LCIMs—translational, rotational, strain and mixed—and their impact on LCIMs functionality, and the next section will further discuss these motions that can be utilized in certain promising applications.

## Applications of LCIMs

**Figure 5.**
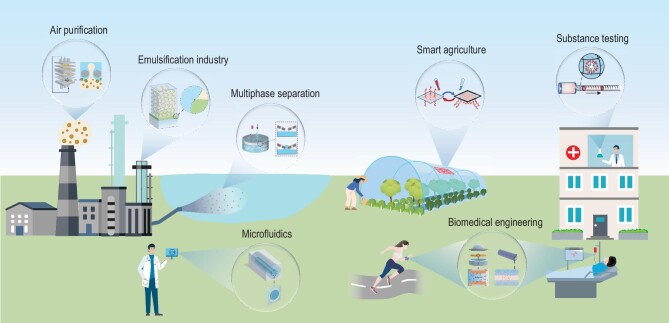
Applications of LCIMs. There are numerous fields in which LCIMs can be utilized, including air purification, multiphase separation, the emulsification industry, smart agriculture, substance testing, microfluidics and biomedical engineering. In air purification, they enable maintenance-free, efficient, low-cost systems with antibacterial, odor and harmful-gas-removal capabilities. For multiphase separation, LCIMs facilitate fine control and dynamic modulation of fluid sorting under steady-state pressure, nearly eliminating fouling. In the emulsification industry, they reduce resistance and energy consumption significantly, producing uniform droplets and protecting temperature-sensitive biological components. LCIMs can also contribute to smart agriculture by smart ‘breaths’ during the heating and cooling cycles of agricultural greenhouses. In substance testing, LCIMs are employed as the core materials of the electroless visual liquid gating detection method, which enables on-site real-time analysis, allowing for rapid detection of metal ions and biochemical quantification. LCIMs can also enhance microfluidics with adaptive systems for stable liquid–liquid interfaces and contamination prevention. In biomedical engineering, LCIMs can enhance medical devices such as catheters to reduce thrombosis and enable targeted drug delivery. They can also be utilized in wearable devices to provide real-time and continuous monitoring of physiological data.

LCIMs show defect-free, responsive and adaptive interface behaviors by utilizing the rich dynamic characteristics of the liquids inside the mesoscopic-scale confined solid porous structures, as well as soft interfaces. This is fundamentally expanding the vision and design spectrum for composite materials, and this idea will play a key role in many fields, such as air purification, multiphase separation, the emulsification industry, smart agriculture, substance testing, microfluidics and biomedical engineering (Fig. 5). The development of LCIMs has also brought about new technologies, such as liquid gating, which was selected as one of the ‘Top Ten Emerging Technologies in Chemistry’ by the International Union of Pure and Applied Chemistry (IUPAC) in 2020 [92]. Next, taking this technology as an example, we will briefly introduce some of the latest progress in the application of LCIMs.

Current mainstream air purification systems face problems such as easy clogging, low efficiency and the short service life of consumables. The LCIM system can effectively remove particles in the gas. By designing electrochemically responsive LCIMs, using the gating liquid as the main filtration material, and further connecting through pipelines, automating and programming control, it is possible to realize the operation of a maintenance-free, efficient and low-cost air purification system [46]. Moreover, the designed gating liquid material portion can endow the purification system with specific purification capabilities, such as being antibacterial and corrosion resistant, and removing odor and harmful gas, providing a new idea for the design of future indoor air purifiers without consumables.

Multiphase separation is an important application in the fields of petrochemicals, water treatment and agriculture engineering. Membrane separation technology is one of the most efficient approaches [93]. However, the permeation of continuous multiphase substances during the membrane separation process often leads to issues like fouling and performance degradation over time, making multiphase separation a considerable challenge [51]. Moreover, multiphase separation often relies on pressure variations, which lead to energy consumption and unstable conditions. To tackle these challenges, an elastomeric liquid-gating technology [44] was developed to finely control and dynamically modulate the sorting of a wide range of multiphase fluids under a steady-state applied pressure, nearly eliminating fouling. This technology also allows multiphase fluids to pass through without clogging, enabling efficient separation processes, such as the separation of emulsions [45,83]. With the responsive and smart design of LCIMs, more applications for multiphase transport and separation control have emerged [55,78,86,94–97]. For example, a magnetic-responsive self-driven system was developed by designing the magnetoelastic LCIMs with reversible meniscus-shaped deformations to realize active regulation of gas/liquid release for visible gas/liquid mixture content monitoring [71].

Emulsification is a critical method for mixing immiscible liquids into droplets in the food and pharmaceutical industries, and in material synthesis. Although commercial emulsification methods hold promise for large-scale production, they suffer from high energy input. The membrane emulsification method offers mild operating conditions, scalability and relatively less energy consumption compared to other methods. Regardless of the number of immiscible liquids in the emulsification process, membrane emulsification initiates at the solid–liquid interface, with the solid matrix imparting the shear stress required to disperse the liquid into droplets. However, due to the large resistance at the solid–liquid interface, this process still requires high pressure. Additionally, contamination or fouling of the surface or interior of the solid matrix is unavoidable. Recently, drag-reducing liquid gating technology has been proposed by using the LCIM to create a smooth liquid–liquid interface for the reduction of resistance and tunable generation of droplets with good uniformity [56]. This technology also provides an appropriate environment for temperature-sensitive biological components (e.g. proteins, enzymes and bacteria) to avoid deactivation by exposure to high temperatures during emulsifying. Compared with existing commercial methods, this drag-reducing liquid gating method can drastically reduce energy consumption, sparking the further development of economically sustainable emulsification applications.

Smart agriculture can improve agricultural production efficiency, reduce costs and promote sustainable agricultural development, which is an important direction for the modernization of agriculture. The issue of thermal control is particularly important in smart agricultural construction, where the heat transfer system, which controls temperature through heating, ventilation and air conditioning, has become one of the major energy issues. Traditional methods mainly involve closed and open systems, both of which have certain limitations in individual heating or cooling processes. Recently, a technology has been proposed that utilizes the LCIM with a bistable interfacial sandwich structure [58]. The temperature responsive LCIM intelligently ‘breathes’ during heating and cooling cycles, dynamically adjusting the indoor temperature to meet the needs of agricultural production. Compared to the closed and open systems, it offers notable energy savings. Applying this system in smart agriculture can reduce energy consumption by 11.6% annually [58]. This new technology holds promise for widespread use in global greenhouse agriculture, paving the way towards achieving carbon neutrality goals.

Substance testing is the process of identifying and measuring the presence, concentration or properties of chemicals in a sample or environment, which plays a crucial role in many fields, such as environmental monitoring, quality control, forensic analysis, medical diagnosis and industrial process control. Techniques employed in substance testing include chromatography, spectroscopy, electrochemical methods, immunoassays and mass spectrometry. The choice of technique depends on the nature of the sample, the target chemicals, the required sensitivity and accuracy of the analysis, and site conditions for application. For instance, it is important for monitoring air, water, soil and other environmental matrices for pollutants, toxins and contaminants to assess environmental quality and protect public health, and in some outdoor environments the passive visualization and portable detections have become very necessary. Progress has been made in the electroless visual detection liquid gating method of chemicals by using LCIMs. This method leverages the dipole-induced interaction between metal ions and surfactant molecules within the specific gating liquids, allowing for the electroless visual detection of metal ions [54]. Recently, this technology has further advanced quantitative detection in biochemistry through the host–guest interactions between gating liquids and target solutions [53]. This technology, based on the LCIMs, simplifies the inherent complexity of sensor design, reduces dependence on specialized equipment, and enhances the potential for on-site real-time analysis. Moreover, it is not only applicable for portable, rapid trace detection of heavy metal pollutants, drugs and biochemical quantification, but also holds broad prospects in fields like food safety, environmental monitoring and medical diagnostics, among others.

The rapid development of microfluidic technology has brought new opportunities to many fields, such as biosensors, pharmaceuticals, chemical analysis and materials science. Fluid control at the microscale level is a promising application, and microscale fluid flow is typically managed either by solid channels [27] or by liquid–liquid interfaces [26]. Nevertheless, the solid channels are plagued with persistent fouling issues, while liquid–liquid interfaces are limited to low-pressure applications [25,98]. To address the above issues, an adaptive microfluidic system prepared by LCIMs has been developed, and it consists of an interconnected porous matrix partially infiltrated with a confined liquid and a microchannel constructed inside [30]. This system is responsive to pressure and enables the formation and instantaneous recovery of stable liquid–liquid interfaces that sustain a wide range of pressures and prevent channel contamination. Furthermore, the confined liquid can also reversibly enter or exit the microchannel by diffusion and wicking into the solid porous matrix, and the process can be further controlled by external stimuli to give this technology extra benefits such as transparency, a liquid gate and self-recovery.

Biomedical engineering is an interdisciplinary field that combines multidisciplinary principles and methods to design and create a wide range of medical devices, diagnostic tools, healthcare technologies, etc. It plays a crucial role in society by providing goods and technologies related to the maintenance and promotion of health. Taking one of the representative medical devices, medical catheters, as an example, these are inserted into the human or animal body for drainage, irrigation, administration, diagnosis or other therapeutic purposes. Conventional catheter materials encounter challenges such as thrombosis formation, limited functionality and insufficient adaptability to environmental changes. To address these issues, a liquid gating catheter has been developed [52]. This catheter not only distinctly reduces the formation of thrombosis, but also enables targeted drug delivery, playing a crucial role in enhancing the safety and functionality of medical catheters [99]. Another example is wearable devices, which can serve as a supplement to diagnostic tools, to provide real-time and continuous monitoring of physiological data. By collecting and analyzing these data, doctors can better understand a patient's health status and make more accurate diagnoses and treatment decisions. In practical applications, the wearable devices often encounter discontinuous states, necessitating the filtration of irrelevant interferences and accurate monitoring of potential health risks. However, most related devices are based on a continuous electrical-signal feedback mechanism. Recently, a discontinuous streaming potential electrokinetic energy conversion system using LCIMs was proposed [85]. This system exhibits discontinuous electrical effects, facilitating gating liquid slip in micropores, with the advantages of gating liquid charge coupling and interfacial drag reduction. Furthermore, this system can not only be applied in autonomous health monitoring devices, but is also expected to be utilized in earthquake and wave warning technologies.

The above development of liquid gating technology will further promote exploration of the functionality and performance of LCIMs. More new technologies and applications based on LCIMs will emerge in the near future.

## Outlooks and challenges

Despite the aforementioned promising application prospects and enormous development opportunities presented by LCIMs and their emerging technologies, many challenges remain, including extending LCIM performance under complex conditions, ensuring their stability and controllability, optimizing the physicochemical matching of materials, and addressing the dynamic interactions of LCIMs with complex fluids. These challenges have limited the opportunities for advancing LCIMs in various applications. Next, we will further delve into some of the challenges that urgently need to be addressed at present.

Firstly, extending the performance of LCIMs even under complex application conditions and promoting their industrial applications should be taken into consideration based on the large-scale and modular design of these materials, as well as their preparation. Moreover, owing to the dynamic nature of liquids, LCIMs are often considered unstable. Thus, to build LCIMs into long-term functional materials, ensuring their controllability and stability will be one of the challenges. In addition, the low volatility of the confined liquid, and the optimal physicochemical matching between the confined liquids and the framework solids, also require further exploration. In particular, when LCIMs come into contact with complex fluids, besides the physical interactions that occur in the compartments, the possible confined chemical reactions that come along will create new requirements for LCIM designs. Added to that, further exploration is needed on the mechanical properties of structural solids, and the optimal physical and chemical matching between the confined liquids and structural solids. For example, sufficient confined liquids can ensure the stable and effective operation of LCIMs, while supplementing or recovering the confined liquids can also improve the economic benefits of LCIMs. The need for constructing smart LCIMs through a combination of responsive structural solids or responsive confined liquids is greater than ever due to the rapid development of intelligent designs in responsive materials. How to improve the stability of responsive LCIMs has become a research focus. This has also made it necessary for LCIMs to have precise and sensitive adaptability to different stimuli: the need for sensitivity to interface tension and viscosity changes of responsive confined liquids, as well as the need for mechanical properties and wetting adjustability of responsive structural solids. Therefore, how to design and prepare controllable, stable and more responsive LCIMs around the key scientific issue of material confined interface control and interaction among solid, liquid and gas (two-phase or multiphase) is an opportunity and challenge for the development of LCIMs. Further breakthroughs are needed in the preparation theory and technical methods of LCIMs to meet the constantly evolving demands of various applications.

Secondly, further research on the transport mechanisms inside LCIMs is crucial for the development of this field. LCIMs mainly include chemically and physically driven modes, as well as their synergistic modes. Taking the membrane system as an example, for the LCIMs with chemically driven transport characteristics, further theoretical research is needed on the mass transfer mechanism at the confined liquid–gas or liquid–liquid interface and the promotion of the transport mechanism within the liquid film. In physically driven systems, the evolution process of confined liquid–gas or liquid–liquid interfaces during fluid transportation, as well as the mesoscopic scale mechanisms that form effective transporting paths, are still unclear. It is necessary to establish simulation and other methods for in-depth research, especially in collaborative modes. A comprehensive understanding of the transport mechanism inside LCIMs will help promote their further rapid development in the future.

Thirdly, the complexity of the confined liquid flows in the soft interface of LCIMs poses difficulties when it comes to understanding the relationships between macroscopic properties and microscopic mechanisms of materials, and it is necessary to combine microscopic characterization methods to conduct in-depth research on the confined liquid interface phenomena of LCIMs. In addition to the common properties of liquids, it is also necessary to delve into aspects such as liquid flow dynamics, the property of being defect-free, molecular level smoothness, adaptability and micro interactions with solids, in order to integrate liquid as a flowing element into the core design of material interfaces and further enrich the understanding of the dynamic performance of two-phase or three-phase interfaces in solid/liquid/gas. In addition, the synergistic designability between the confined liquids and structural solids, as well as the characteristics of mass, momentum, energy transfer and physicochemical reactions at the confined interface, are also future research directions worth further in-depth consideration. Therefore, in order to achieve the optimal performance of LCIMs, it is necessary to develop technologies for real-time monitoring of the composition and rheological behaviors of confined liquids, for example, by using different methods to characterize the morphology and physicochemical information of the confined liquids in real time during the application process of LCIMs (combination of laser scanning confocal microscopy, ellipsometry, interference microscopy and high-precision liquid gating pressure threshold sensing.)

Additionally, the rise of artificial intelligence (AI), machine learning and the material genome initiative have brought new impetus to the development of LCIMs, as they do in other areas. An accurate prediction of rheological and mechanical properties is important for the design of LCIMs for various applications. However, achieving high predicting accuracy with the traditional sequential method requires a large amount of experimental data, which may not be practical in many situations. To overcome these problems, AI has promoted the rapid development of material science in recent years, making it likely the above challenges will be addressed. Recently, we have attempted to explore and propose a Kriging machine-learning model with an active candidate region, which can be smartly updated by an expected improvement probability method to increase the local accuracy near the most sensitive search region, to predict the mechanical and rheological performance of LCIMs with an active minimal size of experimental data [100]. This preliminary attempt in this field provides us with a reference for laboratory optimization and also helps to promote the potential applications of its materials in many fields. But with regard to more practical application scenarios in the future, combining these AI technologies in the research and development of LCIMs to further accelerate the exploration of the confined interactions between solids and liquids will expand the design scope of materials, improve their performance and ensure their stability. This will bring new opportunities for the intelligent frontier application of these materials.
